# The Challenges of Managing Ovarian Cancer in the Developing World

**DOI:** 10.1155/2020/8379628

**Published:** 2020-03-11

**Authors:** Olivier Mulisya, Franck K. Sikakulya, Mbusa Mastaki, Tambavira Gertrude, Mathe Jeff

**Affiliations:** ^1^Department of Obstetrics and Gynecology, FEPSI Hospital, Butembo, Congo; ^2^Department of Surgery, Kampala International University Western Campus, Ishaka, Uganda; ^3^Faculty of Medicine, Université Catholique du Graben, Butembo, Congo; ^4^Department of Obstetrics and Gynecology, Université Catholique du Graben, Butembo, Congo

## Abstract

Ovarian cancer has high morbidity and mortality rates among cancers of the reproductive system. The disease typically presents at late stage when the 5-year relative survival rate is only 29%. Similarly, access to prevention, early diagnosis, treatment, and palliative care for cancer-related disease is insufficient. The availability of cancer treatments in Africa is especially poor. *Case*. A 17-year-old lady, nulliparous, was admitted with complaint of abdominal swelling and loss of weight and a huge left ovarian cyst revealed by ultrasound scan. Laparotomy was done, and a mass which resembled a hemorrhagic solid tumor was found. Grossly, the left ovarian mass measured 15.0 × 20.0 × 8.0 cm and a left salpingectomy was performed. Two months later, she came back with lower limb swelling progressively increased in a week with vulvar edema, with a palpable mass. She was discharged on request by her relatives for traditional medicine. One year later, she passed on in an unrevealed picture. The management of ovarian cancer is too challenging in low-resource countries, from hospital settings to the communities with poor cancer awareness. It is therefore imperative that healthcare resources, policies, and planning focus to be coordinated in a rational way.

## 1. Introduction

Ovarian cancer is recognized as the most leading cause of death among gynecology cancers with a yearly incidence of 239,000 new cases and 152,000 deaths worldwide [1]. Late diagnosis is the main cause of this mortality [2].

Ovarian cancer has high morbidity and mortality rates among cancers of the reproductive system. According to global estimates, 225,000 new cases were detected each year, and 140,000 people annually die from the disease [3].

In Africa, studies have shown that ovarian cancer is the second most common gynecological cancer in developing countries [4]. A woman's lifetime risk of developing ovarian cancer is 1 in 75, and her chance of dying of the disease is 1 in 100. The disease typically presents at late stage when the 5-year relative survival rate is only 29% [1].

Ovarian cancer has a usually relatively poor prognosis; it is disproportionately deadly because it lacks early detection or screening tests implying that most cases are not diagnosed until they have reached late stages. Possible molecular markers including microRNAs, methylation markers, ultrasonography, and computed sonography may facilitate early diagnosis [5].

In about 10% of cases, ovarian cancer tends to occur spontaneously. The key to controlling ovarian cancer appears to be early detection and treatment at the very early stages when cure may be theoretically possible [6].

The global focus to combat cancer needs to be on cancer awareness, early detection, diagnosis, and availability and affordability of treatment in all cancers [5].

The clinic presentation of ovarian cancer has nonspecific symptoms and this lead to its late diagnosis in an advance stage using random abdomen ultrasound scan or laparoscopy [7].

Provision of cancer care is a multidisciplinary effort that necessitates both anatomical pathology and clinical laboratory services. In many parts of sub-Saharan Africa, some oncology services have functioned without the necessary pathology-based diagnosis or laboratory tests that should be offered by pathology departments [8].

Similarly, access to prevention, early diagnosis, treatment, and palliative care for cancer-related disease is insufficient. The availability of cancer treatments in Africa is especially poor [9].

In view of the insufficient attention paid historically to cancer in Africa, the number of cancer specialists as a proportion of all healthcare workers is probably low [9]. Additionally, insufficient resources for pathology lead to inadequate workforce, poor facilities and equipment, and low availability of immunohistology [8].

We are reporting the case of ovarian cancer in a 17-year-old lady for the purpose of showing the challenge in the management of cancers in general and especially ovarian cancer in low settings, in the Eastern DR Congo.

## 2. Case Report

**Figure 1:**
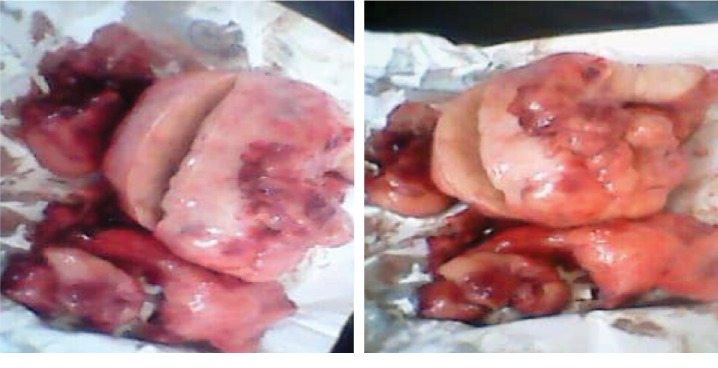
Ovarian tumor resected.

**Figure 2:**
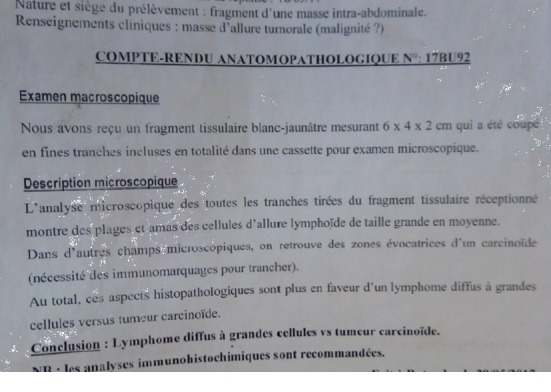
Pathology laboratory results.

A 17-year-old lady, nulliparous, was admitted in the gynecological ward with complaint of abdominal swelling and loss of weight. Her menstrual history was unremarkable. On examination, a palpable abdominopelvic mass was detected; the ultrasound scan revealed a left ovarian cyst (15 × 17 cm), no free fluid was seen, the rest of her abdominopelvic sonographic examination was normal, and the initial clinical diagnosis was one of left ovarian cyst. Her Hb was 10.3 g%; the urinalysis, HIV test, hepatitis, and beta hCG were unremarkable. A laparotomy was planned three days later, and perioperatively, the mass resembled a hemorrhagic solid tumor as shown in Figure 1. Grossly, the left ovarian mass measured 15.0 × 20.0 × 8.0 cm and was brownish and hemorrhagic, with solid and cystic areas, irregular surface with adhesions of the colon to the mass, and no ascites. The right tube and ovary appeared normal with a grossly normal uterus, and a left salpingo-oophorectomy was performed and hemostasis achieved. She was given one unit of blood. The specimen was sent to the laboratory for histology and the result came out after 24 days and revealed diffuse lymphoma with largest cells vs. carcinoid tumor with requirements of doing immunohistochemistry (Figure 2). Her postoperative findings were unremarkable, and she was discharged on her 6^th^ postoperative day.

**Figure 3:**
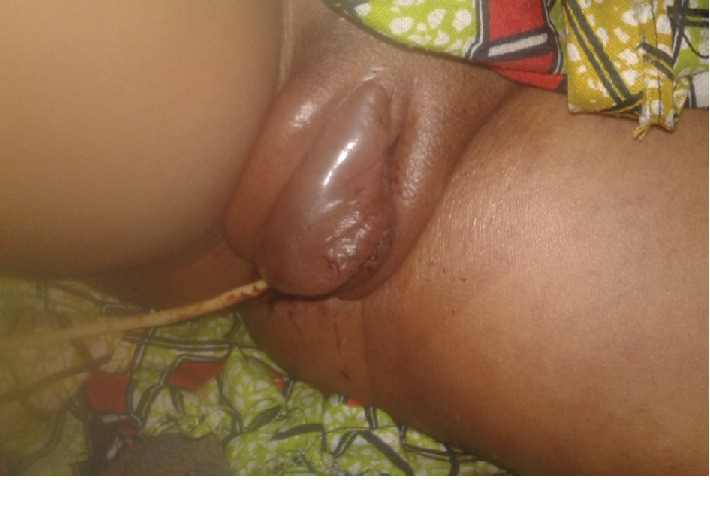
Lower limb and vulva swelling.

She came for review a month later without any complaint, her hemoglobin was 13.2 g%. Two months later, she came back again with lower limb swelling progressively increased in a week as shown in Figure 3. She has received ibuprofen and cloxacilline as an outpatient unsuccessfully. Her last menstrual period was 21 days back.

On examination, she was in a fair general condition, alert, afebrile on touch, no pallor, no jaundice with general edema, and no palpable lymph nodes. Her weight was 42 kgs. Her blood pressure was 90/60 mmHg.

The abdomen was not distended, but with palpable mass, firm and tender in the hypogastrium.

The vulva was edematous with lower limb swelling.

The laboratory tests revealed:

- Urea: 60.95 mg/dl, creatinine: 10.5 mg/dl (ten times higher than the normal range)
- Serum hCG was negative
- Hemoglobin: 10.4 g/dl
- WBCs: 3,700

The ultrasound scan revealed a mass of 11.6 × 9.6 cm in the hypogastrium with bilateral hydronephrosis.

The diagnosis of metastatic ovarian cancer was made with renal failure.

She was given IV hydrocortisone 200 mg three times a day with 2.5 l of normal saline and IV lasix 80 mg. The urine output was monitored, and oliguria was noticed despite the dose of lasix.

She was given IV ceftriaxone 1 g two times a day and IM diclofenac 75 mg two times a day.

The patient did not improve and had persistent abdominal pain, lower limb swelling, and oliguria despite the kidney challenge and her treatment. On day 8, her relatives requested for her to be discharged for further management in Beni which is close to their home.

Later on, we heard that the patient was given some traditional medicine, and the patient improved well and went back to a training center where she was learning hairstyle. For a year, she was doing somehow with progressive weight loss as reported by her relatives.

On a bad note, we heard that she was taken to the general hospital and was managed as an outpatient but passed on two days after the check up in an unrevealed picture.

## 3. Discussion

We presented a case of a 17-year-old lady who died within one year of presenting with recurrent abdominopelvic mass associated with weight loss. The clinical findings as well as histology let us believe that this could be a case of ovarian malignancy.

In this report, we described a case of ovarian cancer which was difficult to confirm without immunohistochemistry and to direct the proper management. It is very clear that this patient needed adjuvant chemotherapy immediately after the first surgery and probably radiotherapy but these could not be available despite the final diagnosis.

There is a high incidence and mortality from gynecological cancers in developing countries due primarily to the failure of these countries to mount effective nationally organized screening programs. A huge unmet need for funding for cancer care and control exists in low-resource countries [6]. Hospitals in Butembo have no pathology department; specimens are sent to the capital city, 2000+ miles through the Université Catholique du Graben's laboratory for reading; CA125 tests are not available and not affordable. There is no cancer unit around. There is no oncologist in all regions. Besides all these, chemotherapy is also not available. And radiotherapy is nowhere to be seen in the whole country.

The management of cancer requires a specialist's knowledge; there is a paucity of all types of specialists (oncologists, pathologists, radiologists, etc.). Not only are specialist resources scarce but also are support services and essential medications lacking in our region.

Policymakers need to understand that cancer care is a comprehensive endeavor that relies on the ability to properly classify the specific type of cancer (and optimally identify other phenotypic and genotypic characteristics that affect prognosis and guide treatment) and on the availability of tests that can be used to follow response to treatment and to detect tumor recurrence [8].

One of the main reasons for the high cancer mortality in sub-Saharan Africa is poor public knowledge and awareness about cancer. Cancer awareness is especially important to improve risk reduction behaviors, promote timely cancer screening for early detection, and ultimately reduce the cancer burden in sub-Saharan Africa [9]. The patient in this case report was taken for prayers and traditional medicine because most people still believe in witchcraft for some diseases quite challenging like cancers.

The main factors causing low cancer awareness in sub-Saharan Africa are the political environment, the economic situation (including funding support), societal norms, cultural beliefs, and values [9]. All investigations are not affordable from histology to treatment of cancers in the region. For our case, the relatives could not afford the 250 (USD) requested for immunohistochemistry. And even for chemotherapy or radiotherapy, they would have had to cross the border to the nearest country Uganda for further management.

During surgery, only inspection, adhesiolysis, and unilateral salpingo-oophorectomy was done, yet according to Schorge et al. [10], peritoneal washings should be collected immediately upon entering into the abdomen, followed by exploration. The ovarian mass should be removed intact and submitted to pathology for frozen section evaluation. However, it is almost impossible to know with certainty whether a patient has a benign adnexal mass, low malignant potential tumor, or invasive ovarian cancer until final histologic slides have been reviewed. In those with low malignant potential tumors diagnosed intraoperatively, limited staging biopsies of the peritoneum and omentum should be considered. Additionally, the appendix also should be examined and potentially removed, especially if the tumor has mucinous histology. Routine pelvic and para-aortic lymph node dissection is also considered in the presence of enlarged nodes or a frozen section suggestive of frankly invasive disease [10].

## 4. Conclusion

The management of ovarian cancer is too challenging in low-resource countries, from hospital settings to the communities with poor cancer awareness.

It is therefore imperative that healthcare resources, policies, and planning focus to be coordinated in a rational way.

Advocacy and the political will to invest in the development of human resources and healthcare infrastructure appear critical to gynecological cancer control and reducing the burden of disease in our region.

A strong coalition between governments, experts, communities, and donor agencies will be needed to achieve these goals.
